# Structured human-LLM interaction design reveals exploration and exploitation dynamics in higher education content generation

**DOI:** 10.1038/s41539-025-00332-3

**Published:** 2025-06-18

**Authors:** Pablo Flores Romero, Kin Nok Nicholas Fung, Guang Rong, Benjamin Ultan Cowley

**Affiliations:** 1 Faculty of Educational Sciences, University of Helsinki, Helsinki, Finland; 2 Cognitive Science, Faculty of Arts, University of Helsinki, Helsinki, Finland

**Keywords:** Education, Human behaviour

## Abstract

Large Language Models (LLMs) present a radically new paradigm for the study of *information foraging behavior*. We study how LLM technology is used for pedagogical content creation by a sample of 25 participants in a doctoral-level Artificial Intelligence (AI) in Education course, and the role of computational-thinking skills in shaping their foraging behavior. We used editable prompt templates and socially-sourced keywords to structure their prompt-crafting process. This design influenced participants’ behaviors towards *exploration* (to generate novel information landscapes) and *exploitation* (to dive into specific content). Findings suggest that exploration facilitates navigation of semantically diverse information, especially when influenced by social cues. In contrast, exploitation narrows the focus to using AI-generated content. Participants also completed a Computational Thinking survey: exploratory analyses suggest that trait cooperativity encourages exploitation of AI content, while trait critical thinking moderates reliance on participants’ own interests. We discuss implications for future use of LLM-driven educational tools.

## Introduction

Education evolves along with the technologies available to it. However, the mere adoption of novel technology does not ensure favorable outcomes. To realize the full potential of the newest artificial intelligence (AI) technologies, it is essential to understand their impact on pedagogy and how educators integrate them into psychological and instructional practices^1,2^.

Among recent innovations in AI, Large Language Models (LLMs) such as OpenAI’s Generative Pre-trained Transformer (GPT) model^3^ represent a major breakthrough for content generation, answering questions, brainstorming, among many other tasks. This breakthrough has sparked widespread discussions on the opportunities and challenges it presents within the field of AI in education (AIEd)^4–11^.

However, due to the novelty of this technology, AIEd research has only begun to explore the cognitive and psychological mechanisms of human-LLM interactions in educational settings. *It remains an open question: how do educators’ personal information processing and computational thinking traits impact their use of LLMs?*

Here, we examine this open question in an experimental study using two theoretical frameworks: Information Foraging Theory (IFT)^12–14^ and Computational Thinking^15–17^.

IFT provides a valuable structure that can inform and conceptualize navigation in AI-generated information. The Chatbot implementation of GPT (ChatGPT) has contextual memory of each conversation like a human interlocutor, but unlike a human, can be asked to generate a fresh conversation at any time. Thus, users can both generate fresh content at any time, and dive deeper in a coherent way by prompting for additional details, thus enriching a given content with more information. This dynamic closely mirrors the two fundamental information foraging decisions: *Exploration*, where agents search new information landscapes for value (GPT generates fresh content and we read them), and *Exploitation*, where agents utilize a valuable landscape to its full potential (we ask GPT to further elaborate on an interesting content). Drawing on these principles, IFT’s model of foraging decisions provides a systematic framework to explore and understand the ways human navigate information and sensemaking processes within digital environments^18–21^.

Although ChatGPT can interact quite like a human, it remains an algorithm. The human user’s *computational thinking* skills might therefore modulate their interactions with ChatGPT. It remains unclear exactly how this would work because ChatGPT is not a *programmable* algorithm, but possibility is demonstrated in principle by work on ‘prompt engineering’^22,23^. In a seminal contribution,^15^ Wing underscored the importance of Computational Thinking skills, which enables people to solve tasks in a similar way that computer algorithms work and are beneficial across many professional domains. Since then, computational thinking skills have gained importance in digital education research, and some work suggests that they correlate with a more confident and efficient use of digital technologies^16,24,25^. In Human-AI interactions^26^, reported a significant correlation between computational thinking skills and the determinants of AI literacy, which encompass the knowledge to use, recognize and evaluate AI-based tools^27^. also found, through a controlled experiment, that using ChatGPT in a programming course improved the computational thinking skills of their students. Thus, while computational thinking skills appear to influence and be influenced by human-AI interactions, precise mechanisms remain to be uncovered.

In summary, our study uses IFT and computational thinking frameworks to explore how relevant cognitive and psychological traits of educators govern their interaction with an LLM.

The **study design** was within-subjects, where we conducted a controlled interaction between doctoral candidate educational scientists (taking a course on AIEd) and ChatGPT (model GPT-4). We gathered pre- and post-interaction data: before the course, participants described their AIEd interests; after the course, they submitted interaction-inspired assignments and answered a computational thinking survey.

The study was conducted within an on-campus doctoral course titled ‘Basics of AI in Education’ in the years 2023 and 2024. Participants were assigned the task of developing their own study designs to investigate hypothetical scenarios involving AI applications in educational settings, co-creating these hypothetical scenarios with ChatGPT. This approach both enriched personalized content and introduced participants to this novel tool.

From participants’ AIEd interests gathered before the course we derived a set of topical keywords to inform our interaction design. Halfway through the course, each participant completed a 45 min GPT session to create personalized scenarios, guided by a researcher (2023: PF, 2024: KF) who mediated between participants, our interaction design and ChatGPT. Over the following weeks of the course, the participants were asked to: first, design a hypothetical study and research plan based on their chosen scenario, and second, write a reflective essay based on the scenario and planned study (they were not expected to implement the study). As participants were trainee educational scientists, we anticipated that their assignments would follow their personal research interests (reported at the beginning of the course), but this was not required by us. To minimize influence on decision making, researchers followed a predefined protocol (see Methods).

**Fig. 1:**
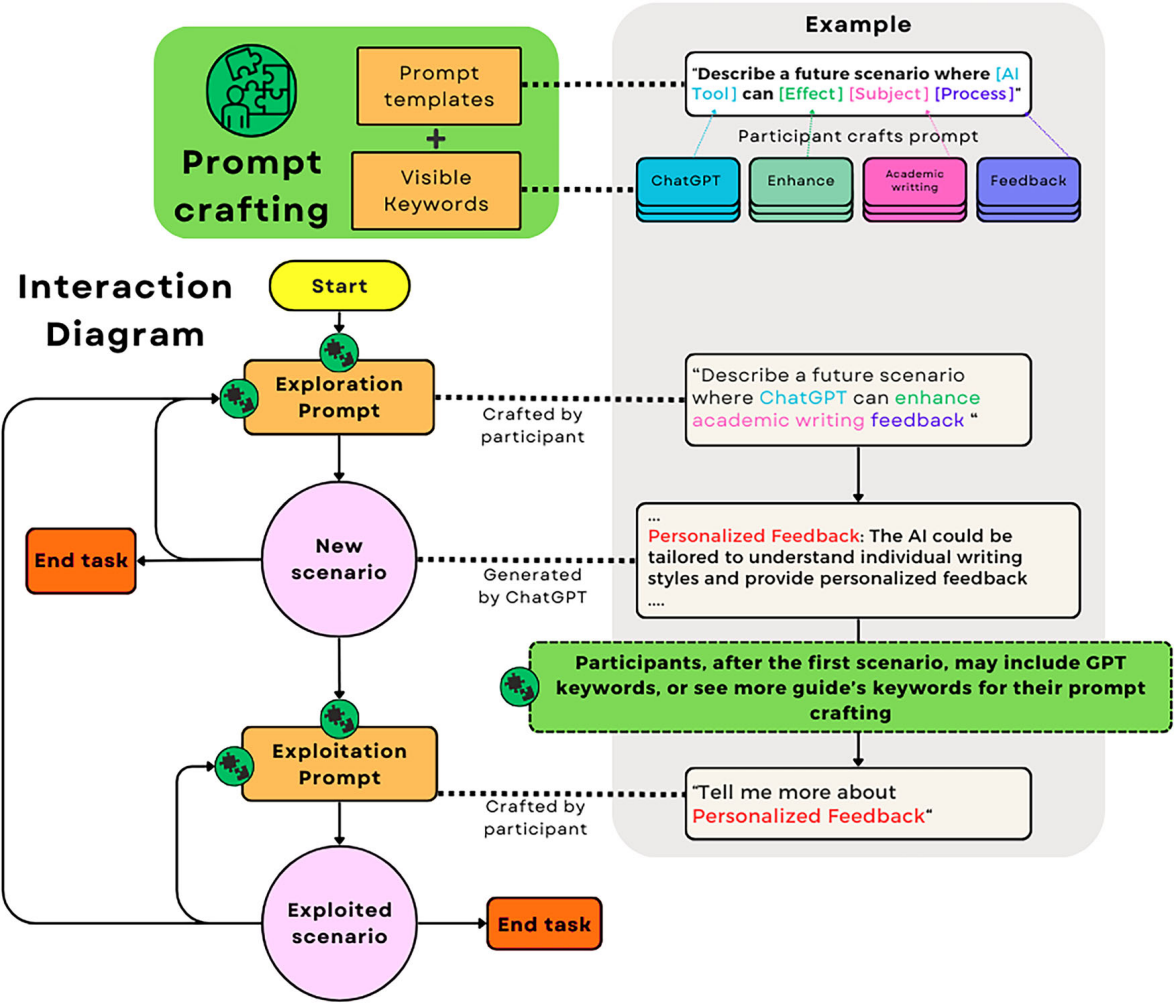
Interaction diagram. Participants craft their prompts by selecting a prompt templates and keywords of interest. The process initiates with an *Exploration* prompt, to generate a hypothetical assignment scenarios. Subsequently, participants can select new templates and keywords, and craft prompts to *explore* new scenarios, *exploit* interesting scenarios, or end the task by choosing a scenario that is interesting for them. After viewing the first scenario, participants may view additional guide keywords or include GPT keywords into their prompt crafting. Note: The examples provided are simplified; actual prompts and ChatGPT's responses are more detailed.

Our **Interaction design** aimed to facilitate participants’ shared task goal—creation of personalized content—in a standardized study setting amenable to quantitative analysis. We developed a novel interaction protocol, illustrated in Fig. 1 and described below.

Our interaction design aimed to systematically measure human decision making when foraging in novel AI-generated information landscapes. The interaction design allowed participants to generate personalized content through the use of editable *prompt templates* (designed by us) populated by a set of *keywords* (derived from an early course assignment). Specifically, we wanted to observe when participants were generating entirely new information (*exploration*) versus when they were diving deeper into a particular concept or idea (*exploitation*). To do this, we provided two types of *prompt templates*. **Exploration prompts** were used whenever participants decided to generate a completely new scenario, marking the start of an independent chat with ChatGPT (ensuring no context carryover from previous interactions). We counted each use of these prompts as an instance of *exploration*. **Exploitation prompts** were used when participants wanted more details about a concept or idea in the context of an already generated scenario. We counted each use of these prompts as an instance of *exploitation*. See Fig. 1 for brief examples of each prompt type and Supplementary Materials for the full details. These *prompt templates* facilitated the *exploration-exploitation* behavioral modes of IFT, standardized the interaction between participants, guided those without prior experience, and enabled systematic analysis of their foraging behavior.

The *set of keywords* allowed us to observe how participants’ interests and self-representations shape their foraging. This followed from Cowley et al.’s^28^ concept of a socially-shared virtual environment for crowd-sourced parameter optimization of user-oriented AI. Furthermore, to explore the influence of AI on the foraging behavior of the participants, they had the option to use keywords sourced from AI-generated information. With these elements (codified as per Table 1, see Methods for details), participants crafted prompts tailored to their interests, which were used to create (explore) and elaborate (exploit) their personalized scenarios in ChatGPT.

**Table 1:** Behavioral variables of participants’ interactions with GPT

Code	Description
Exploration	N^∘^ of exploration prompts used
Exploitation	N^∘^ of exploitation prompts used
Viewed Kw	Max. rows of keywords participants
	managed to see during the interaction.
Guide’s Kw	N^∘^ of used keywords sourced
	from the design
GPT Kw	N^∘^ of used keywords sourced
	from the GPT-generated text
Own Kw	N^∘^ of used guide’s keywords that the
	participant provided to the design
Other’s Kw	N^∘^ of used guide’s keywords that the
	participant did not provide to the design.
Time (mins)	Duration in minutes of the interaction, measured
	from the first prompt to the last.

We also **analyzed using topic modeling** the course assignments submitted by the participants. These texts were analyzed to measure their topic diversity and explore potential correlations with their information foraging behavior. For this analysis, we used the BERTopic framework, a data-driven algorithm that employs BERT-based embeddings along with clustering and representation techniques to extract interpretable topics from text data^29^, and used the Shannon Index^30^ to quantify topic diversity, measuring how broad or narrow their topical scope was.

Lastly, following Celik's and Yilmaz & Karaoglam Yilmaz's^26,27^ work, we employed the Computational Thinking Scale (CTS) at the end of the course. Developed by Korkmaz et al.^31^, CTS is a self-report scale defining computational thinking as the result of 5 sub-factors, Creativity, Algorithmic Thinking, Cooperativity, Critical Thinking, and Problem Solving.

**In summary**, with this research design, we explored two main research questions. First, *how do educators explore and exploit AI-Generated information with GPT in a pedagogical content generation task*? Second, *how do educators’ computational thinking skills—encompassing creativity, algorithmic thinking, cooperativity, critical thinking, and problem solving — manifest in their interactions with GPT*?

This report covers two iterations of the study. We first collected data in 2023 and shared our preliminary results^32^. Encouraged by these results, we collected more data during 2024 and performed the same analysis. In addition, we expanded our discussion to include the AIEd context and introduced a new dimension: analyzing text data for topical diversity. This allowed us to explore the semantic variety in participants’ interactions and beyond. Although the results from the second cohort supported and enriched our previous findings on the dynamics of exploration and exploitation, they do not match our first results^32^ regarding the influence of CTS factors on these dynamics. Recognizing the heterogeneity within our sample as a potential cause of this failure to find the same effect, we performed a cluster analysis on the demographics of our participants to allow for an intra-group examination of the impact of CTS factors on foraging dynamics.

## Results

We collected data from 25 GPT interactions, matched with 25 CTS surveys: 9 in 2023 and 16 in 2024. The interactions lasted from 4–43 min (M = 21.88 min, SD = 11.06), involving one to six prompts (M = 3.04, SD = 1.34). A total of 76 prompts and 232 keywords were analyzed. In addition, we processed 22 essays (3 missed from 2024) and 25 study designs to assess topic diversity. From this, we identified patterns in prompt use, keyword selection, and their association with the participants’ topic diversity and the results of the CTS survey.

### Exploration and exploitation

The results show that the frequency of exploitation prompts was similar to exploration prompts (Table 2 and 3), with ten participants using more exploitation than exploration prompts. Fifteen participants explored only one scenario, nine explored two scenarios, and only one person explored three scenarios. Only one participant stopped at the minimum allowed number of actions (generating and selecting one scenario without further prompting).

**Table 2:** Descriptive statistics of behavioral variables

(*n* = 25)	mean	median	sd	min	max
Exploration prompts	1.44	1.00	0.58	1.00	3.00
Exploitation prompts	1.60	1.00	1.38	0.00	5.00
Viewed keywords	1.96	2.00	0.54	1.00	3.00
GPT keywords	1.12	1.00	1.51	0.00	6.00
Guide keywords	8.16	7.00	3.57	4.00	18.00
Own keywords	2.64	2.00	2.10	0.00	7.00
Others keywords	5.52	5.00	2.79	2.00	13.00
Conversation duration [min]	21.88	21.00	11.06	4.03	43.00

**Table 3:** Total use of prompt types and used keywords

Prompt type	Total uses	Prompts with	Prompts with
	(% of total)	Guide’s Kw (%)	GPT Kw (%)
Exploration	36 (47%)	36 (100%)	0 (0%)
Exploitation	40 (53%)	17 (42.5%)	24 (60%)

One Exploitation Prompt Was Constructed with a Mix of Guide and GPT’s Keywords.

*Notes:*1. Percentages in the “Total uses” column are relative to the sum of all prompts.

2. Percentages in the “Prompts with Guide’s/GPT Kw” columns are relative to the total uses of the respective prompt type.

In contrast, there is more variability in the use of exploitation prompts. Although optional, 19 out of 25 participants (76%) used at least one exploitation prompt. Participants who explored only once used more exploitation prompts (*M* = 1.8, SD = 1.3) than participants who explored more than once (exploitation *M* = 1.2, SD = 1.5); the difference was not significant (Mann-Whitney *U* = 51.5, *p* = .18). We also observed that the use of exploitation prompts was more frequent in the selected scenarios than in the ones discarded (Table 4).

**Table 4:** Use of exploitation prompts in selected and discarded scenarios

Scenario	Total exploitation	# of Scenarios	# of Scenarios with
	prompts used (%)	exploited (%)	GPT Kw (%)
Selected (*n* = 25)	38 (95%)	19 (76%)	14 (56%)
Discarded (*n* = 11)	2 (5%)	1 (9%)	1 (9%)

*Notes:*1. Percentages in the “Total exploitation prompts used” column are relative to the total number of exploitation prompts across all scenarios.

2. The percentages in the columns “# of Scenarios Explored” and “# of Scenarios with GPT Kw” columns are relative to the number of scenarios within each category (25 for Selected, 11 for Discarded).

### Choice of keywords

Separating by prompt type, exploration prompts exclusively incorporated guide’s keywords, whereas exploitation prompts mainly employed GPT keywords identified within the generated scenarios (Table 3). Notably, 14 out of 19 participants that used exploitation prompts used at least one GPT keyword with them.

Lastly, Table 4 shows a predominant presence of both exploitation prompts and GPT keywords within the selected scenarios, contrary to the discarded ones.

Regarding the use of the guide’s set of keywords, we found that, overall, most of the guide’s keywords used by participants corresponded to other’s keywords, not personally provided by the participant, instead of their own (138 out of 204, 68%). All participants included others’ keywords in their prompts. Regardless, we also observe that most participants, except for five, included at least one of their own keywords in their prompts. Regarding the tone of the scenarios, defined by the use of keywords from the ‘effects’ category, the majority of the participants specifically inquired about positive scenarios (n of the participants = 17) instead of negative scenarios (*n* = 4), or a combination of them (*n* = 4).

### Topic modeling

We extracted topics from three sources of text data: participant interactions (through 199 keywords), essays, and study designs. Table 5 summarizes the collected data from the essays and study designs, which were preprocessed and decomposed into sentences. The number of identified topics and the topic diversity of the participants, calculated as a Shannon diversity index, are shown in Table 6.

**Table 5:** Data description of essay and study design documents

Metric	Essay	Study design
**Document Information**		
N# of documents	22	25
Document’s character length	1350.45 ± 300.76	1644.72 ± 923.90
**Sentence Metrics**		
N# of sentences	1160	1712
Sentences per document	55.55 ± 15.47	75.56 ± 40.15
Words per sentence	25.47 ± 12.35	23.80 ± 11.73

Note: values presented as *mean* ± *SD.*

**Table 6:** Number of topics identified and Shannon index for participants’ topical diversity across different types of text data

Metric	Essay	Study Design	Keywords
N# of topics	23	34	21
Shannon entropy	1.78 ± 0.37	1.70 ± 0.45	1.66 ± 0.29

Note: values presented as *mean* ± *SD.*

The results of the keywords informed us about their foraging content and diversity in semantic terms. In general, the participants used a wide range of concepts that resembled their diverse backgrounds and research interests. However, most of the topics identified were present in several participants and few were unique to individual participants. Examples of broadly shared topics include learning (*n*_*participants*_ = 17), teaching (*n* = 12), and higher education (*n* = 10). The identified topics in the keywords encompassed mostly, but not exclusively, groups of synonyms or very similar words. For example, the topic of ‘learning’ included the keywords ‘learning’, ‘enhanced collaborative learning’, ‘learners’, ‘answering questions’, and ‘individual learning’, among others.

When comparing keyword sources, the guide keywords covered a broader range of topics, while the GPT keywords were more context-specific. In particular, the topic with the highest representation in the GPT keywords, representing 28% of them, contains keywords related to analysis such as ‘Data analysis’, ‘Tracking feedback’, ‘Monitoring Progress’, and others. The same topic was underrepresented in the use of guide keywords.

The results of topic modeling for assignment data allowed us to examine the nature (assessed qualitatively) and diversity (assessed as entropy of topic representation, see Methods) of topics beyond interaction. Although the topics aligned with the course and the conceptual contents of the keywords, the nature of the identified topics had three different types. Firstly, there was one broad and ambiguous topic, representing the general theme of the course (AIEd), but not being precise about any specific approach. This topic contained the highest number of sentences in both assignments (20% for essays and 16% for study designs). Secondly, there were more defined and specific topics, typically each associated with an individual participant, reflecting their unique perspective (e.g., emotional learning or creativity in the essays). Lastly, there were specific topics that appeared in several participant texts (e.g., ChatGPT and academic writing in the study designs), indicating common areas of interest within the course. Further details on topics can be found in the supplementary information (See Supplementary Tables 1,2, and 3).

We identified two significant correlations between the participants’ foraging behavior during the interaction and their topic diversity. First, there is a significant positive correlation between the frequency of exploration prompts and the diversity of keywords used by participants (*τ* = 0.57, *p* = 0.0008). This holds for the use of guide keywords (*τ* = 0.57, *p* = 0.0002), and is similarly explained by the use of their own keywords (*τ* = 0, 37, *p* = 0.02) and the keywords of others (*τ* = 0.34, *p* = 0.02). Second, there is a significant negative correlation between the frequency of GPT keywords and the topic diversity in the essays of the participants (*τ* = − 0.47, *p* = 0.01). Notably, this correlation does not extend to the use of exploitation prompts, suggesting that this pattern might be unique to the exploitation of GPT-generated information.

### Associations with CTS

For the total combined data from 2023 and 2024 participants, no significant relationships were identified between behavioral variables and CTS factors, failing to match earlier published work^32^.

Taking into account the heterogeneous nature of our sample’s demographics and how these might relate to the studied variables, we employed a clustering procedure to obtain sub-groups of participants (see Methods). Specifically, we considered demographic variables age, gender, nationality, educational level, experience with GPT, and years of teaching experience. Three more homogeneous groups were identified, mainly distinguished by their age, nationality, and years of teaching experience (Table 7).

**Table 7:** Identified groups of participants, based on demographic clustering

Group	Age	Years of teaching	Nationality
(size)		Experience	
1 (*n* = 10)	29.1 ± 2.28	2.0 ± 2.79	Diverse (6 Finns)
2 (*n* = 8)	40.12 ± 5.06	2.38 ± 1.85	Diverse (3 Finns)
3 (*n* = 7)	42.43 ± 2.57	15.86 ± 4.56	Only Finns

Note: values presented as *mean* ± *SD.*

**Fig. 2:**
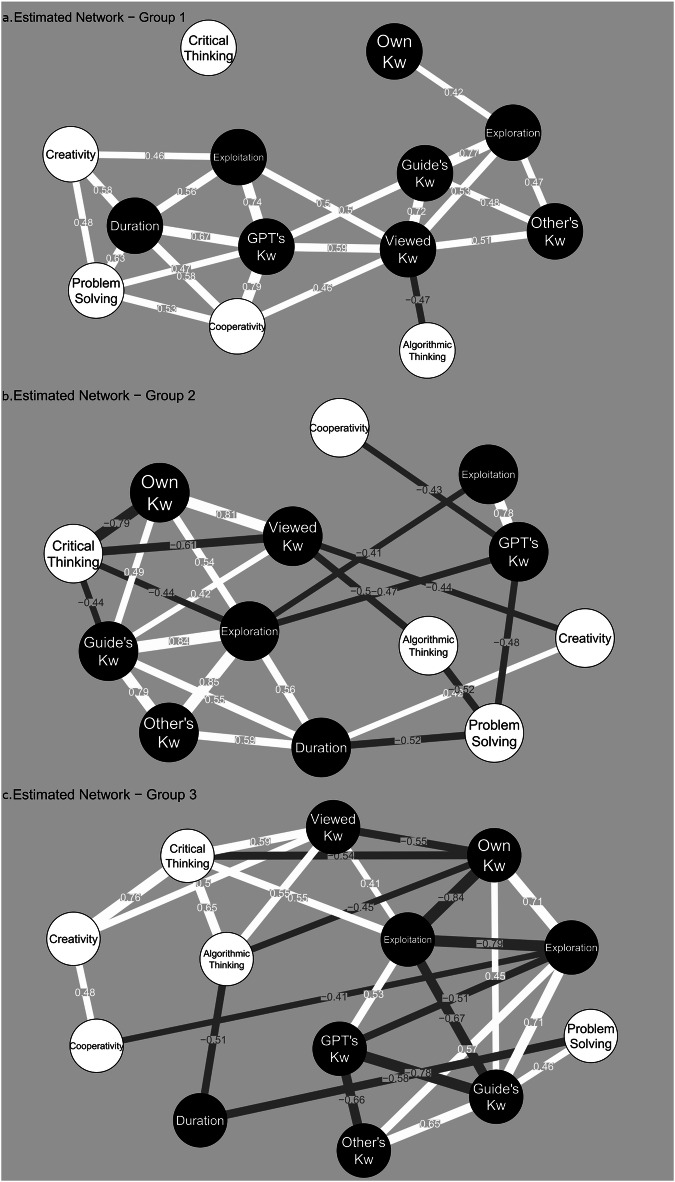
Network analysis. Estimated network structures between the behavioral variables and the Computational Thinking Survey’s (CTS) sub-factors' scores, per group. Panel (**a**) for group 1 (*n* = 10), panel (**b**) for group 2 (*n* = 8), and panel (**c**) for group 3 (*n* = 7). Nodes symbolize variables: Black for Interaction Behaviors (Kw Keywords), White for CTS sub-factors. Edges represent Kendall’s tau correlations: White for positive, Dark gray for negative. Spurious edges are filtered based on a strength threshold set at *τ* > 0.4.

Figure 2 shows the relations between the behavioral variables and the CTS’s sub-factors, per identified group. The bootstrapped CIs showed significant relationships within groups 1 and 2.

For Group 1, the results indicated a significant positive correlation between the amount of GPT keywords used and their scores in cooperativity (*τ* = 0.79, *p* = 0.01), with bootstrapped CIs ranging from 0.56 to 0.94.

For Group 2, there was a significant negative correlation between the use of their own keywords and scores in critical thinking (*τ* = −0.79, *p* = 0.01), with bootstrapped CIs ranging from -1.00 to -0.24. A similar negative correlation was observed in group 3, but did not reach statistical significance (*τ* = − 0.54, *p* = 0.11).

## Discussion

Our exploratory study examined educators’ information foraging behavior when using GPT in an pedagogical content creation task, and the associations with their assignments’ topic diversity and their computational thinking skills. Study results answer our research questions, showing that:

- Exploration is influenced by socially-sourced keywords
- Exploration keywords have higher topic diversity, i.e., these prompts were conceptually diverse
- Exploitation is influenced by GPT-sourced keywords
- Exploitation keywords have lower topic diversity, i.e., these prompts were conceptually narrower
- Exploitation is associated with less-diverse essay topics
- CTS associations with GPT interactions are heterogenous across sample demographic clusters

Prior research in digital information foraging emphasizes the roles of *information scent* and *social cues* in guiding user behavior. *Information scent* refers to the perceived value of information in relation to a user’s goals, shaping how individuals navigate digital environments^33–36^. Social cues, such as rankings, recommendations, and forums, further enhance foraging efficiency and shared sense-making^19,36–40^. These factors provide a useful lens for interpreting the findings, particularly in understanding how participants navigated through the information in regard to their own interest.

The **exploration prompts** were exclusively constructed using the guide’s set of keywords derived from participants’ reported interests (Table 3). We also observed that participants frequently opted to include keywords contributed by others, rather than their own, in their exploration prompts.

This suggests a deviation from a commonly-advocated thesis of *personalized learning*^41,42^, in which participants’ own interests would drive their foraging behavior. Instead, we observed that the interests of others, encapsulated in the shared keywords, played a major role in framing their exploration prompts.

Sharing information scents contrasts to one issue of exploration in AI-generated information: in the absence of predefined structures, users may not be exposed to alternative views^43^. In our study participants did not have this limitation because they encountered diverse topics and perspectives via keywords from classmates, making their exploration more robust.

Thus, when designing pedagogy around AI-generated information, where predefined information structures are absent, social cues can have value for the prompting process. Shared elements could include frequently-used concepts (keywords), common verb types or sentence structures (prompt templates), prompt ratings, records of positive/negative previous outcomes; any elements tied to prompt crafting. Such design elements could foster a constructivist and shared sense-making of generative tasks and learning goals, mitigate the risk of echo chambers, while providing educators with quantitative insights into students’ interests, focuses, and performance in human-LLM foraging tasks.

Our **topic modeling** results revealed that the use of exploration prompts and the guide’s (i.e. socially-sourced) keywords effectively increased the diversity of topics within the participants’ interactions. This increase supports our conceptualization of exploration prompts as mechanisms for navigating semantically-varied information landscapes. In addition, the analysis identified trending topics, such as shared interest in ChatGPT, alongside individual focuses seen in topics dominated by single participants. Identifying these trends can provide insight into whether class and individual interests converge or diverge on specific topics. These insights could further inform the development of pedagogical strategies that balance personalized content with collaborative learning opportunities.

This finding suggests a meaningful social process, where educators explore pedagogical information while influenced by a collective conceptual space gathered from the keywords, rather than being driven solely by personal interests. In other words, sharing cues among peers enables educators to innovate their content creation processes, broadening the diversity of learning content.

For **exploitation of information**, we observed a distinct process that highlights how AI-generated content influenced the crafting of prompts. These prompts concentrated in the selected scenarios (Table 4), suggesting that participants deliberately chose these scenarios over others, recognizing their added value for task completion (i.e., they where interest for the course assignments). This observation aligns with the IFT definitions, where exploitation is viewed as a means to utilize and enrich an informational landscape to its fullest^13,35,44^.

Regarding the keywords used in the exploitation prompts, we found that there was a preference for using GPT keywords (see Table 3). This shift suggests that, as the interaction progressed, participants relied more on AI-generated information for scents to follow, diverging from the guide’s keywords guidance. However, it is important to note that this shift still occurred within the context set by the initial exploration prompt. The specific and contextual nature of the exploited GPT keywords helped refine and specify elements within generated scenarios.

Our **topic modeling** results suggest that an increased use of GPT keywords is associated with a lower topic diversity in participants’ essays. Notably, this correlation was exclusive to the exploitation of GPT keywords, as it did not transfer to the use of exploitation prompts in general. Assuming that the topic diversity in the essays reflects the breadth or depth of participants’ reflective focus, this finding raises several hypotheses: First, participants confident in a specific topic, with a narrower focus in their essays, might be more inclined to exploit the highly-contextualized GPT keywords. Conversely, those with a less specialized focus might be less interested in exploiting GPT keywords, resulting in greater topic diversity. Alternatively, GPT keywords may influence participants’ mindsets towards narrower topics. Regardless of the underlying cause, this phenomenon resonates with the IFT conceptualization of exploitation as a form of focusing efforts by narrowing the scope toward what is identified as valuable.

Interestingly, in two cases, a used GPT keyword matched a participant’s reported interest, underscoring the adaptability of GPT in tailoring content to user preferences. This observation aligns with the theoretically proposed capabilities of LLMs in adaptive learning^5,6,10,45^. However, this also raises a concern. Unlike the guide’s keywords, which can be validated during the design process, AI-generated content requires real-time critical assessment by users. This is particularly difficult in educational environments, where students are still developing expertise. Subhash^46^ showed that LLMs can have a strong persuasive influence over user preferences, while Amoozadeh et al.^47^ observed that many programming students used LLMs to directly answer problems and avoid effort, even under supervision.

In summary, exploitation prompts may not only add value to AI-generated content, but this value may be largely derived from specific scents identified within the AI-generated information landscape. By generating specific information scents for educators to follow and exploit, which derive value from elaborating on the pedagogical themes driving the interaction, LLMs might hold potential in enhancing educational foraging tasks.

**Exploration and exploitation** co-exist in a trade-off relationship. Barke et al.^48^ explored this dynamic by examining *how* programmers interact with the GPT-based Copilot assistant. Using qualitative grounded theory analysis (not an IFT analysis per se), they identified a bimodal usage pattern in Copilot’s adoption. Programmers alternate between *exploration*, where hesitant users employ Copilot as a planning assistant to suggest structure, and *acceleration*, where confident users employ Copilot to speed up code authoring in small units.

Our findings align with these insights, revealing a similar *exploration-exploitation* trade-off in a content creation task. Participants balanced between *exploration*, driven by the broad conceptual space of the interaction design, and *exploitation*, guided by the specificity of the AI-generated text. While *exploration* facilitated broad content generation through a socially shared conceptual space, *exploitation* enabled deeper navigation into specific pieces of AI-generated information. Notably, our study also revealed an interplay between individual and peer interests in shaping this balance. While AI-generated cues guided individual later navigation, peer-sourced keywords enriched the initial exploratory process. This contrasts with *personalized learning* strategies^41,42^, which typically adapt content based on individual preferences alone. Instead of predefined tailoring, our approach empowered participants to make choices within a socially enriched conceptual space, fostering greater topic diversity during exploration.

These findings highlight the role of social elements in educational settings, an aspect often overlooked by the individual-centric focus of current AIEd systems. This highlights a broader tension between socialized and individualized learning models in AI-driven education. Intelligent Tutoring Systems (ITS) emphasize personalization by profiling learners and tailoring content to their competencies^41^. The global push for personalized learning has led to algorithm-driven, results-oriented strategies that risk disconnecting from educational theory^49,50^. In contrast, our findings suggest that balancing personalization with social exploration-rather than focusing solely on individual optimization-can foster richer learning experiences.

Building on this balance between personalization and social exploration, educational theories have long stressed the importance of integrating both breadth and depth in learning strategies. Studies suggest that a balanced approach-combining expansive exploration with targeted depth-optimally supports learning needs^51,52^. In the context of LLM based systems, educators should be cautious of overemphasizing one aspect at the expense of the other. Furthermore, long established learning theories highlight the importance of socially enriched learning paths. Vygotsky’s^53^ zone of proximal development and mediation, which highlights the importance of structured guidance in expanding learners’ capabilities, while Bandura’s^54^ social learning theory highlights how peer interaction shapes knowledge acquisition. By incorporating both scaffolding and social elements, AI-driven educational tools can foster richer, more adaptive learning experiences.

*Summarizing the first IFT research question*, our findings emphasize the role of social elements in guiding exploration during AI-generated foraging tasks, while the shift toward exploitation highlights the potential and risks associated with relying on AI-generated keywords. These insights provide initial steps for understanding user behavior in AI-generated information landscapes, which is key to developing intuitive, safe, and user-centered AI systems.

Regarding **computational thinking skills**, our results provide mixed evidence for their influence on LLM interaction (i.e. we did not match earlier work^32^). LLMs adapt well to different tasks without requiring extensive re-design of the model’s architecture and do not require advanced technical programming skills to use. Thus, users from different domains can now rapidly tailor their interactions to align with their contexts and needs, predominantly via the construction of prompts to elicit artificially generated content^9^.

Prompts, being essentially natural language instructions to an LLM, are amenable to prompt engineering methods deployed in the back end to enhance LLM performance, as measured through technical benchmarks^22,23,55–58^. These methods are varied, including prompt chaining, self-improvement, task decomposition, among others. They enable a pre-trained model to adjust their behavior—for example, shifting between exploration and exploitation. Can such purposive prompt construction be achieved by users at the front end? Answering this requires us to examine and understand how human cognitive mechanisms like computational thinking could contribute to effective interaction with LLMs.

Here we have asked whether users’ prompt construction naturally reflects the cognitive faculties they would need to *program* a computer?^32^ showed that algorithmic thinking and creativity played a role in dissociating exploration and exploitation modes. However, we did not match these results directly. Instead, our network analyses revealed two potential trends in more homogenous sub-samples.

First, we observed that among participants in group 1, characterized as younger with less teaching experience and diverse nationality, favorable scores on cooperativity might promote the use of GPT keywords in their foraging behavior. In the CTS, cooperativity construct is an approach to learning, in which the respondent seeks to maximize learning of individual and group members^31^.

Second, in group 2, characterized as middle-aged without extensive teaching experience, higher critical thinking scores were linked to a reduced use of their own keywords in the task. This pattern was also observed in group 3, consisting of Finnish participants of similar age and much more teaching experience, but not in group 1. CTS approaches critical thinking as an active, regular, and reflective process focused on deciding what to do and what to believe^31^. Based on this, we hypothesize that critical thinking could potentially influence the scent detection process, leading participants to allocate more cognitive resources to evaluate the different keywords rather than relying heavily on their reported interests.

These findings suggest that demographic factors such as age and cultural background might interact with individual cognitive traits such as cooperativity and critical thinking, to be relevant in how participants engage in human-AI interactions during co-creation tasks. Although several studies have explored human-GPT collaborative dynamics, they have often overlooked sample demographics^59–63^.

A recent study ^64^analyzing factors that influence the acceptance and use of ChatGPT found that age, education, and employment substantially impact the use of technology. This may explain the observed differences among our groups and suggests that controlling for age, educational qualifications, and cultural background in larger samples might provide further insight into this phenomenon.

Another interpretation for the lack of significant patterns in the pooled data considers the novelty effects of the tools. Groups 2 and 3 are composed of either all (100%) or a majority (62%) of 2024 participants, who had an additional year of exposure to LLMs. This longer exposure to ongoing practical and ethical discussion about AI integration in society may have helped them develop experience and confidence in engaging with LLMs and AI-generated content.

**Limitations** of our study include the small sample size, and the lack of qualitative methods to complement our quantitative focus. While we increased the sample size compared to previous research, it remains relatively small for robust multivariate analysis. This constraint limits the generalizability of our findings, particularly for the network analysis, which would benefit from a larger, more representative dataset. To enhance the robustness of our results, we applied bootstrapping to strengthen the interpretation of the network structure. Future research should validate these findings with larger, more diverse samples to improve reliability and applicability. Based on the general rule of requiring five observations per independent variable^65,66^, a minimum of 200 human-LLM interactions (with full prompt sequences) would be necessary for confirmatory studies.

Additionally, although it incorporates text data for topic modeling, our study relies heavily on quantitative analysis. Therefore, we were unable to fully capture the reasoning behind participants’ decision-making and could only hypothesize about the underlying factors behind, for example, the influence of social cues during exploration or GPT’s keywords during exploitation. A good example of how mixed methods might enhance such insights is provided by Barke et al.^48^, where qualitative analysis of semi-structured interviews and field observations was corroborated with quantitative analyzes of the task.

Finally, our results do not reflect typical use of ChatGPT. While our design involves the use of ChatGPT, our focus is not on modeling the typical conversational style of user interactions with ChatGPT. In typical use, ChatGPT allows for free-form prompting, where users can craft and refine their queries as they wish, often leading to dynamic, open-ended conversations. In contrast, our study employed a structured interaction design, intentionally limiting participants’ freedom to prompt at will.

Our template-keyword design allows modifications for different purposes, our specific task complicates comparisons with previous web search studies that formally assess IFT principles. To rigorously evaluate IFT in human-LLM interactions, future designs should align with analyzed web search tasks, such as the one described by Kittur et al.^38^. Further studies contrasting socialized and individualized approaches for LLM-based interaction designs could provide valuable insights for AIEd future development, particularly in personalized content generation. By tailoring AI generated content to shared interests or organizing group foraging around common topics while also supporting individual exploration^28^, educational scientists might better navigate in generative AI learning environments.

**I**n **conclusion**, our results show the potential of structured prompting interfaces to inform us about both *information foraging dynamics* within AI-generated landscapes and the development of thoughtful *educational interaction* designs.

Participants balanced between *exploration* and *exploitation*, finding a trade off between the broad concepts of the interaction design and the specific AI-generated concepts, respectively. We see that social elements may play a crucial role in the early exploratory stages of information foraging tasks with LLMs, while AI-generated information facilitated deeper individual navigation. Moreover, we see that the role of computational thinking varies and may be linked to demography. These insights can guide the design of effective pedagogical tools to leverage the potential of LLMs for educational purposes.

## Methods

### Study design

This exploratory study observed and analyzed participants using LLMs, where interactions were controlled within a structured, theory-driven framework. Rather than testing specific hypotheses, we designed the interactions to foster participant-driven foraging of AI-generated content. The methodology reflects a single-arm, quasi-experimental study design, which introduced a structured but flexible task as a class intervention, but did not employ random assignment or control groups. This approach enables systematic examination and identification of emergent patterns in participants’ behavior.

### Setting and participants

We conducted this study during two iterations of the doctoral course called “Basics on Artificial Intelligence in Educational Sciences” at the University of Helsinki. Participants were recruited using a convenience sampling approach from enrolled students. Although they were not financially compensated, cinema tickets were provided to all participants who gave their consent to participate in the study.

The study was carried out in accordance with the Declaration of Helsinki, with the ethical approval of the Research Ethics Committee in the Humanities and Social and Behavioural Sciences, University of Helsinki (27/2022). Informed consent was obtained from all participants.

The participant pool (*n* = 25) ranged in age from 24–52 years (mean = 36.36), and while culturally diverse, the majority were Finnish (*n* = 16). The group had 17 women. Most participants were Ph.D. students (*n* = 20), while the rest were M.A. students. All had fluent English skills. They were all specialists in Educational Sciences, but their approaches were diverse and included cognitive sciences (*n* = 2), technology education (*n* = 3), psychology (*n* = 4), early childhood education (*n* = 2), science education (*n* = 2), among others. Participants’ experience with ChatGPT varied; most (*n* = 17) had prior exposure, while a minority (*n* = 9) had none.

### Procedure

Due to a lack of established framework, we developed this novel procedure to investigate human-LLM interactions. In the design, we captured the interaction of each participant with GPT through a semi-structured co-creation process, allowing us to standardize and frame their decision-making in terms of exploration and exploitation. To ensure a smooth task performance and consistent quality across participants, two authors (PF and KF) facilitated the sessions, guiding participants through the co-creation process. Participants booked 45 min sessions with the researchers, though no strict time limits were enforced during the activity.

To provide adequate task guidance while preserving student agency and decision-making, we established the following protocol:

- **Setup:** Prompt templates were displayed on a table along with the keywords cards, face down in 5 rows, divided by the colored categories.
- **Introduction:** Participants were briefed on the aim of the task-creating an interesting scenario for their assignments-and introduced to the use of prompt templates and keywords, including an example to illustrate their application. Usage information was reminded during the interaction to ensure effective performance with the task.
- **Execution:** When the aim and rules were clear, we start a timer and participants began by uncovering the first row of keywords, selecting a prompt template and keywords of interest to craft a prompt. We then input the prompt to ChatGPT, read the generated text and participants chose their next option: to generate (explore) new scenarios, to further elaborate (exploit) the current one, to view more of the guide’s keywords, or to end the task by choosing the scenario for their course assignment. We also highlighted the option of including GPT keywords to explore or exploit scenarios. If the participant crafted a new exploration prompt, a new conversation with ChatGPT was opened, to remove the previous information from the context. Participants were reminded of all their options when required.
- **Conclusion:** Once the participant decided that a scenario was interesting enough for them to end the task, we stop the timer and provided them with the full transcript of the conversation for the selected scenario.

After their co-creation task with GPT, the participants had ~1 month to submit their assignments through an online platform (Moodle). Following the assignment deadline, participants completed the CTS survey via an online platform (REDCap), distributed through email.

### Materials

Our **interaction design** consists of six editable *prompt templates*, divided in two types; and a set of *keyword cards* distributed unevenly in seven different categories, representing different concepts contextualized within the course topics. All of these elements were printed and laminated. We used ChatGPT-plus with the latest versions of GPT-4 (as of May 2023 and May 2024).

The prompt templates were divided between *Exploration* and *Exploitation* prompt types. *Exploration* prompts were designed to create the assignments’ scenarios through a fixed imperative sentence, like “*Predict me a future scenario where*…” or “*Describe me a scenario where*…”. In the subsequent editable part of the prompt, participants could combine from four to six keywords to shape the scenario to their interests. *Exploitation* prompts were designed in a similar fashion, but focused on a created scenario, following structures like “*Tell me more about this scenario, but put more emphasis on*…”. The subsequent editable part was restricted to one or two keywords. While the templates defined the action behind the prompt (Exploration or Exploitation), their keywords defined the specific and desired content to generate.

The design used a categorized pool of keywords shared between participants that were contributed by them at the beginning of the course (See Supplementary Fig. 1). The keywords’ categories were roughly defined beforehand, based on a general set of concepts that were able to define the core constituents of AIEd scenarios, like ‘Actors’, ‘AI tools’, ‘Environments’, and ‘Subjects’. Eighty-six keywords were contributed in the first iteration of the course and 95 in the second. Additionally, we included six keywords in an ’Effects’ category, divided equally in positive (e.g., benefit, enhance) and negative (e.g., hinder, disrupt) effects.

The **computational thinking scale** was developed by Korkmaz et al.^31^. CTS is a self-report scale based on the framework provided by the Computer Science Teachers Association (CSTA) with the International Society for Technology in Education (ISTE)^67,68^, and the Computational Thinking Leadership Toolkit^69^. The scale consists of 29 items divided in the corresponding 5 sub-factors–Algorithmic Thinking, Cooperativity, Creativity, Problem Solving and Critical Thinking. Each of the items in the factors has been scaled as never (1), rarely (2), sometimes (3), generally (4), always (5).

Given that the original scale was developed within a computational science department, we adapted three items to better suit our educational settings (see Table 8). To verify the internal consistency and reliability of the adapted survey, we conducted an initial analysis using Cronbach’s alpha and McDonald’s omega and assessed the coefficients when items were dropped. This analysis revealed that several items affected the overall reliability. In consequence, we removed seven items (I59, I57, I56, I66, I26, I21, I20) due to low item-total correlations or their negative effect on the scale’s consistency. In particular, items I59, I57, I56, and I66 had low factor loadings in the original validation study. Furthermore, items I26, I21, and I20 were strongly framed in a mathematical manner, which might explain their inconsistency with the other items in our context.

**Table 8:** Adaptations of the computational thinking scale (original by Korkmaz et al., 2017)

Item	Original item	Adapted item
I26	"I can immediately establish the equity	"I can quickly construct the equation which
	that will give the solution of a problem”	would yield the solution to any problem”
I27	"I believe that I can easily catch the	"I believe that I can easily catch the relation
	relation between the figures”	between symbols and conceptual figures”
I57	"Dreaming causes my most important	"My dream is an important factor
	projects to come to light”	when I perform many tasks”

Following the removal of these items, the revised scale showed improved reliability in both the aggregated score (Cronbach’s *α* = 0.87, McDonald’s *ω* = 0.92) and in the subfactors: Algorithmic Thinking (*α* = 0.82, *ω* = 0.94), Cooperativity (*α* = 0.88, *ω* = 0.93), Creativity (*α* = 0.72, *ω* = 0.86), Problem Solving (*α* = 0.65, *ω* = 0.83) and Critical Thinking (*α* = 0.84, *ω* = 0.89).

### Data analysis procedure

**Fig. 3:**
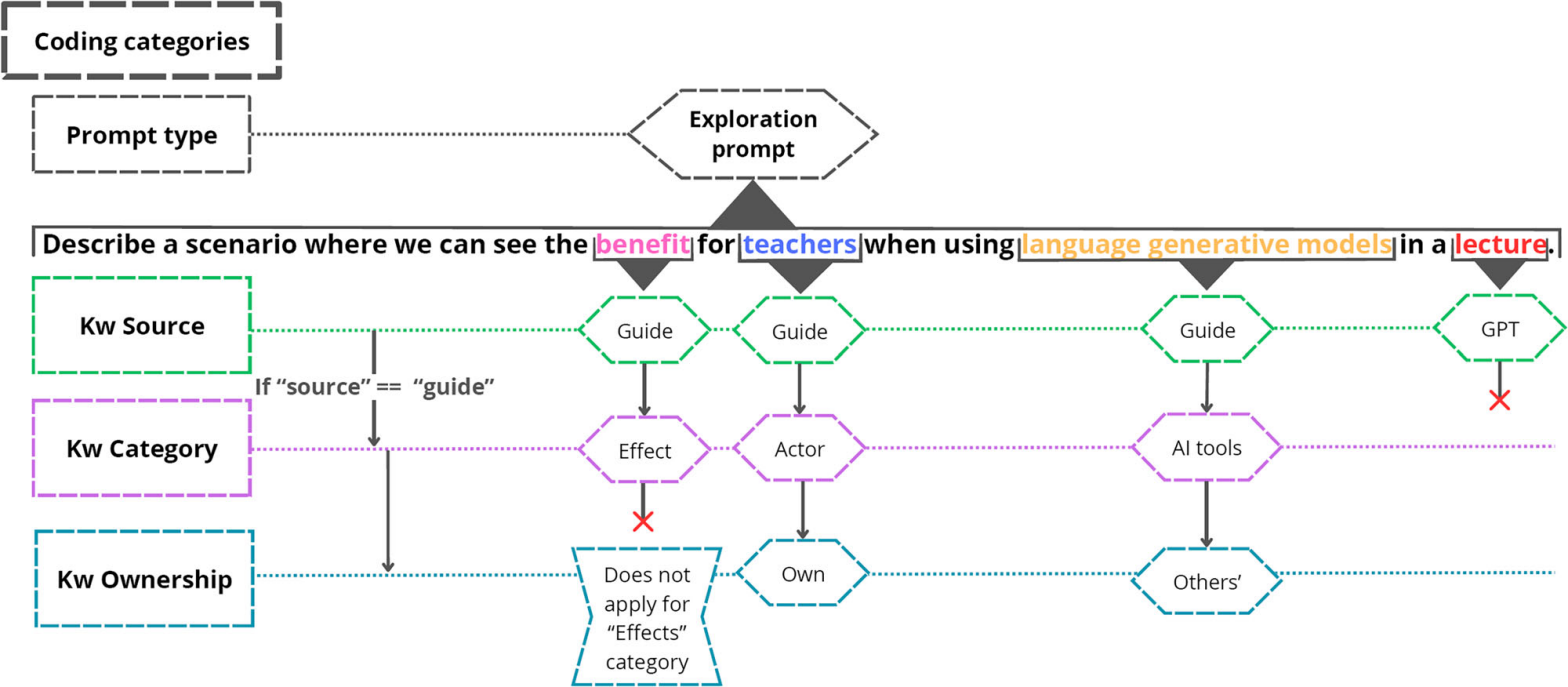
Coding strategy. Prompt types (exploration or exploitation) contained the keywords categories. Keywords were coded, in order, by source (Guide or GPT); category (defined by the design); and ownership (own or others' keywords). GPT keywords were not coded in any category due to its dynamic nature.

Behavioral data from participant interactions was processed and coded, as shown in Fig. 3, to capture the frequency and co-occurrence of behavioral variables (see Table 1). Additionally, Prompts were also classified based on the use of positive or negative ’Effects’ keywords to determine which were more prevalent. Descriptive statistics were used to evaluate behavioral patterns in prompt construction.

The assignment and keyword text data was analyzed using the BERTopic^29^ topic modeling framework to extract topics participants interactions and assignments. Shannon diversity index^30^ was then calculated to quantify the topic diversity of participants in assignments and interactions, followed by a correlation analysis to explore potential relationships between behavioral variables and topic diversity.

The participants were clustered according to demographic features. For each cluster, network analysis was used to explore the associations between the eight behavioral variables and five factors. Network results were qualitatively compared across clusters.

### Statistical methods

To assess **behavioral patterns**, participant-level data was presented as mean ± Standard Deviation (SD) (see Tables 2, 5, 6, and 7). Overall observations were presented as frequencies and percentages of occurrence and co-occurrence (see Tables 3 and 4).

The **topic modeling and diversity analysis** was built on the BERTopic framework, involving five distinct and modular stages: First, assignments were stripped of bibliography sections, segmented into sentences and converted into **embeddings** using the ‘all-MiniLM-L6-v2’ BERT-based model^70^. Keywords were embedded without segmentation.

**Dimensionality reduction** was applied to these embeddings using Uniform Manifold Approximation and Projection (UMAP) with 15 neighbors, 5 components, and a minimum distance of 0. **Clustering** was performed using Hierarchical Density-Based Spatial Clustering of Applications with Noise (HDBSCAN), with two parameter configurations. For the assignments, we set a minimum cluster size of 10 and a minimum of five samples. For keywords, we set a minimum cluster size of 5 and a minimum of 2 samples. Both procedures used Excess of Mass (eom) cluster selection method. A **bag of words** was generated by first using an English vectorizer, removing stop-words and allowing for bi-grams in the representations. Then we performed a class-based TF-IDF with BM-25 weighting and square root term frequency procedure, and **fine-tuned** the topics using the KeyBERT model^71^.

Non-categorized sentences, constituting approximately one-third of the data, were assigned to the closest topic embeddings using cosine similarity, while irrelevant topics (inline citations) were removed from the analysis. The topic embeddings were calculated as the average of their respective sentence embeddings.

We proceeded to arrange the data by creating class-based data-frames for each participant, as well as by keyword source (i.e., Guide’s/GPT Kw). These data-frames captured the topics identified, and the number of sentences per topic, for each participant and keyword source.

Finally, the Shannon diversity index^30^ was used to quantify the topic diversity within each document, using the relative frequencies of topics within each document and a base-10 logarithm.

**Kendall’s tau correlation** test was employed to handle non-normal data, tied ranks, and small sample size. This test was used both to assess correlations between behavioral variables and topic diversity, and to construct the network analysis.

For **demographic clustering**, we selected demographic variables of age, gender, nationality, educational level, experience with GPT, and years of teaching experience. We recoded nationality to *Finnish* or *non-Finnish*, and education level to *Master’s student* or *PhD researcher* (some of which were part-time and also working as lecturers). No rows had missing values. Based on iterated k-means clustering over *k* = [1, 10], the elbow of the within cluster sums of squares metric indicated a three-cluster solution. We then applied t-distributed Stochastic Neighbour Embedding (tSNE) algorithm to partition participants, with dimensions 2, perplexity 8, theta 0.5, and 500 iterations. t-SNE obtained error <0.15, sparsity 0.96, and three clusters which were robust over multiple repeats of the algorithm.

To conduct **network analysis**, the relationships between the behavioral and CTS variables were represented as an undirected network. The variables were represented as nodes, and the connecting edges reflected the calculated Kendall correlations. A minimum correlation strength threshold of 0.4 was applied to filter out edges, and 2500 non-parametric bootstrapped samples were used to calculate 95% Confidence Intervals.

### Rationale

Our study was designed to systematically include complex behavioral observations, surveys, and quantified text data. Thus, each data point (participant) is high-dimensional and comes from lab-controlled conditions that limit variation, which lends more power to our analysis. Although the sample size is small, this drawback is partly ameliorated by the richness of the data. This helps us to infer structure between variables (as with, e.g. longitudinal designs), computed using bootstrap-based network analysis.

Importantly, we are perhaps the first study to look at information foraging indicators in LLM-human interaction, which means we lack prior evidence to compute a sample size; our study provides a basis for future studies to compute sample size.

To assess **behavioral patterns**, our coding framework was developed to capture the different decisions available to our participants within the interaction design. They included the prompt type, the source of the keyword, the keyword category, and the ownership of the keyword (see Fig. 3). However, we excluded keyword categories from the analysis due to their ambiguous boundaries, with the exception of the ‘Effects’ category, which exhibited a clear distinction between ‘positive’ and ‘negative’ sentiments. By examining the co-occurrences of these decisions, we aimed to identify behavioral patterns within each prompt type (exploration and exploitation).

For **topic modeling**, we chose BERTopic due to its adaptability to small data sets and embedding-based nature, which is critical to capture contextual nuances in our limited sample size^72^.

The embedding model ‘all-MiniLM-L6-v2’ was selected for its balance between speed and performance, running locally, and faster than larger models while maintaining high efficiency for text clustering tasks^73^. This sentence-transformer model maps sentences and short paragraphs to a 384-dimensional dense vector space. Unlike LLMs such as Llama or GPT, which are designed to generate text, sentence-transformers are specifically trained to produce embeddings for information search, retrieval, and clustering. To fit the model’s context window of 256 tokens, the documents were decomposed into sentences. This decomposition also allowed for a larger sample of embeddings, and thus increased the effectiveness of the data-driven method. However, due to limited data volumes (*n* < 100), we were unable to extract distinct and coherent topics from the reported interests of the participants (introductory assignments) or their complete prompts (template with keywords). Two topics were removed from the analysis, as they represented inline citations and lacked meaningful semantic information. These were topics 9 (for study designs) and 20 (for essays).

The flexibility of the BERTopic framework allowed for iterative fine-tuning to enhance topic coherence and relevance. Specifically, we adjusted the HDBSCAN parameters to identify more specific topics for the more numerous and semantically similar essays and study designs data by using lower values for minimum samples. For the prompts’ keywords, both minimum samples and cluster size were reduced to capture coherent and distinct topics in a smaller data set.

Class-based data-frames were then constructed, allowing us to examine the distribution of topics across different dimensions, particularly by participant (using the Shannon index) and by the source of keywords. These data-frames proved instrumental in analyzing how topics varied both between participants and across different document types.

Lastly, to quantify how broad or narrow their texts were, we used the Shannon diversity index^30^ to calculate the topic diversity for the documents of each participant. Originally developed to measure entropy in text, this index has been extended to various fields, such as ecological diversity measures^74^ or the assessment of cultural diversity in text^75^. Shannon is a suitable choice for our analysis because it accounts for both the richness (number of topics) and evenness (distribution of topics) in each document. Furthermore, unlike other indices, such as Simpson’s, Shannon is more sensitive to the presence of rare topics^76,77^. This makes it ideal for our data, as it downplays the influence of the dominant topic, which represents the broad and ambiguous theme of the course, and highlights the smaller and well-defined topics.

To analyse **associations with CTS**, considering the exploratory purpose of this study, network analysis was selected due to its versatility and visualization capabilities to explore multivariate relationships holistically^78^. To account for the heterogeneity in our sample, demographic clustering was conducted to form more homogeneous groups for the network analysis.

We applied bootstrapping to strengthen the interpretation of the network structure within a small sample size^79,80^. To minimize spurious edges, we filter based on the correlation strength with a lower threshold of *τ* > 0.4, based on^81^ guideline for moderate correlation strength in psychological studies. Our discussion focused on findings with stable bootstrapped results.

Given the exploratory nature of this study, we did not apply ***post-hoc*** **corrections**, such as the Holm-Bonferroni method^82^, for multiple comparisons. The primary objective was to uncover new insights in a novel context, rather than to confirm any specific hypotheses. Adjusting for multiple comparisons can increase the risk of Type II errors, potentially obscuring meaningful findings. However, the use of network analysis and bootstrapping methods helped us better visualize and filter our multivariate analysis to focus on potentially important effects. Therefore, we prioritized a comprehensive examination of the data, over strict control of family-wise error rate, more pertinent in further confirmatory studies.

### Analytical tools

We used ATLAS.ti 23, a qualitative analysis software, to construct the frequency-based dataset of behavioral variables and derive co-occurrence results. Documents were pre-processed and segmented into sentences using a pre-trained Punkt sentence tokenizer for English, available in the Natural Language Toolkit (NTLK) library. For topic modeling, we utilized the BERtopic implementation in Python (https://github.com/MaartenGr/BERTopic). Shannon’s entropy calculations, correlation analysis, and Network Analysis techniques were performed within the R environment. Network analysis procedures were performed using the *bootnet* package^83^. Network visualization was possible using the *qgraph* package^84^.

## Supplementary information

Supplementary Information

## Data Availability

The datasets gathered and analyzed during the current study are available from the corresponding author upon reasonable request.

## Code availability

The underlying code and scripts for this study are available from the corresponding author upon reasonable request.
