# Supplementary material for: Structured human-LLM interaction design reveals exploration and exploitation dynamics in higher education content generation
Source: NPJ Sci Learn. 2025 Jun 18;10:40. doi: 10.1038/s41539-025-00332-3 (PMC12177052; doi:10.1038/s41539-025-00332-3)
Supplement: Supplementary file 1 — Supplementary Information [file 41539_2025_332_MOESM1_ESM.pdf]

Supplementary Information

This document further supplementary information for: the prompt templates, with examples of use; description of the introductory assignment, with a subset of the resulting keyword cards; the instructions for the course assignments; and topic modeling results related to study designs, essays, and prompts' keywords.

Prompt Templates

Prompts are divided by type. They were printed and laminated. Colored parts represent the editable sections of the prompts and below each one we provided an example of use. Editable parts of exploitation prompts could be filled with keywords from any category, or with GPT keywords.

Exploration Prompts

Describe a scenario where we can see the Effect for Actors / Ed. process when using AI tools in a Environment + Subject + Activity

Describe a scenario where we can see the benefit for teachers when using language generative models in a university language class.

Predict a future scenario where AI tools can Effect + Actors / Ed. process in a Environment + Subject + Activity

Predict a future scenario where language generative models can improve feedback in a high-school language class.

Tell me a different scenario ← Create alternative scenario if the first answer is too boring.

Exploitation Prompts

Tell me more about this scenario but put more emphasis on [element of interest]

Tell me more about this scenario but put more emphasis on feedback

According to this scenario, specify the importance of considering the "[element of interest]"

According to this scenario, specify the importance of considering the "feedback"

In this scenario, can you contrast between [element of interest] and [element of interest - contrasting]

In this scenario, can you contrast between students and teachers.

Examples of use

The following examples were generated during the iterative process of constructing the prompts. Keywords were chosen on demand, guided by intuition about expected outcomes.

"Describe me a scenario where we can see the benefit for teachers when using language generative models in a university language class."

Imagine a university language class where the teacher uses a language generative model to assist students in improving their writing skills. The model is programmed to provide feedback on grammar, spelling, syntax, and vocabulary, and can even suggest alternative phrasings for students' written work.

As the class progresses, the students begin to use the model as a tool to improve their writing. They find that the model's feedback is helpful in identifying areas where they need improvement, and the suggestions provided by the model help them to refine and polish their writing.

The teacher is also able to use the language generative model to customize their teaching approach to individual students. For example, the model can be set up to provide more advanced feedback to students who are more advanced in their language skills, while providing simpler feedback to those who are just beginning to learn.

In this scenario, we see the benefits of using language generative models in a university language class. The model can help students to improve their writing skills, provide personalized feedback, and allow the teacher to customize their teaching approach to individual students. Additionally, the model can save the teacher time by automating the feedback process, freeing up time for other instructional activities.

“Describe me a scenario where we can see the danger for writing when using language generative models in a university language class.”

Imagine a scenario where a university language class uses a language generative model to assist students in writing essays. The model is trained on a large dataset of existing essays, and is designed to generate coherent, well-written sentences and paragraphs.

However, as the students begin to use the model, they realize that it has a tendency to produce overly generic or formulaic writing. The model lacks creativity and originality, and the essays produced by the students all begin to sound the same.

Furthermore, the use of the model can lead to a dangerous homogenization of writing styles and ideas, stifling creativity and individuality in the students' writing. The students may become overly reliant on the model, neglecting the importance of developing their own unique voices and ideas.

In this scenario, we see the dangers of using language generative models in a university language class. While these models can be powerful tools for generating text, they can also promote a form of writing that lacks creativity and individuality. This can be especially problematic in academic settings, where originality and independent thinking are highly valued. It is important for teachers to be aware of these risks and to encourage students to develop their own writing skills, using the model as a supplement rather than a replacement for their own creativity and originality.

“Predict a future where language generative models can improve feedback in a high-school language class.”

In the future, machine learning could potentially revolutionize the way feedback is given in high-school language classes. By leveraging large amounts of data and sophisticated algorithms, machine learning could provide more accurate, personalized, and timely feedback to students, improving their learning outcomes and overall academic performance.

One potential use case for machine learning in language classes is the automated grading of student assignments. By analyzing patterns in student writing, machine learning algorithms could provide quick and accurate feedback on grammar, syntax, and

vocabulary usage, helping students to identify areas for improvement and providing them with actionable feedback to help them grow as writers.

In addition, machine learning could help teachers to identify patterns in student performance and provide targeted interventions to help struggling students catch up. For example, machine learning algorithms could analyze student performance on various assessments and identify students who are struggling with a particular concept or skill. Teachers could then use this information to provide targeted interventions, such as extra tutoring or small group instruction, to help these students succeed.

Furthermore, machine learning could be used to personalize learning plans for individual students. By analyzing a student's performance on various assessments and identifying their specific learning needs, machine learning algorithms could create customized learning plans tailored to the student's strengths and weaknesses. This could help students to focus their efforts on areas where they need the most help, improving their overall academic performance and boosting their confidence in their own abilities.

Overall, the use of machine learning in high-school language classes has the potential to revolutionize the way feedback is given and to improve student learning outcomes. With the right tools and support, teachers could use machine learning to provide more effective and engaging language instruction to their students, helping them to develop the skills they need to succeed in their academic and professional careers.

Construction of Keyword cards

Keywords were extracted from participants' first assignment of the course. This was an introduction task, and they were prompted to write their submission based on the following example:

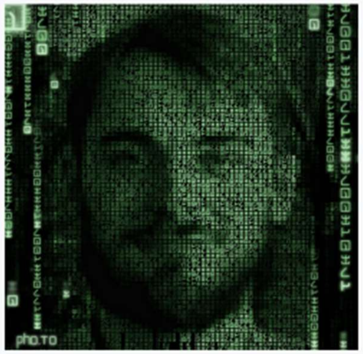

Submit a pdf describing your particular interests in the field of educational sciences, especially regarding AI.
Provide 2 forms of information:

1. You must provide a keyword or phrase for the six categories below:

Actors	AI tools	Subjects	Environment	Activity	Educational processes

For example, Ben could write: "*I am interested in using machine learning [**AI tool**] to study adolescent school [**environment**] students [**actors**], learning [**educational process**] skilled performance in coached classes [**activity**] for primarily visuomotor tasks like sports, games, or music [**subject**]*".
This is an accurate description of one of Ben's genuine research interests. You don't need to copy it, but take it as an example. You can also just supply six keywords for the six categories.
You can supply more than 1 keyword per category, but it's not necessary.

Supplementary Figure 1. Introductory assignment instructions. Prompt given to students through the online platform for the submission of their first assignment.

Supplementary Table 1. Keyword cards. subsample from total keywords, extracted from students' submissions.

Effect	Actor	AI tool	Subject	Environment	Activity	Educational process	
Benefit	Teachers	Large Language Models	Mathematics	Comprehensive school	Classroom interactions with pupils	Professional learning	Teacher's continuous development
Limit	Students	Machine Learning	Education	High school	Community interaction with colleagues	Research	Problem solving strategies
Assist	PhD researchers	Virtual Agents	Calculus	University	Open-ended problems	Analyze eye-tracking data	Tutoring
Disrupt	Researchers	AI-supported reading and writing analysis	Classroom video	Outside academia	Professional vision	Expertise development	Collaborative learning
Improve	Lecturers	AI tutors	Sustainability challenges	Course platforms	Problem solving	Problem-based learning	Learning
Hinder		ChatGPT 4.0	Climate Change	Upper secondary classroom	Interacting with AI	Individual behavioral change	Answering questions

Instructions for Course Assignments

The course had two main assignments, a hypothetical study design and a reflective essay. The instructions provided in the course online platform were as following:

The main aim of your coursework is to examine a hypothetical scenario of AI in education, which should relate to your own research and pedagogical interests (which you were asked to submit in the first week).

You should have created the actual text for your scenario in an interactive session with an AI, a Large Language Model called chatGPT, guided by our research assistant Nicholas.

You must now design a study of this scenario. After submission, you will also get an assignment to write a short essay reflecting on the scenario.

Study design task.

Describe a study of the scenario you created, using a standard scientific report format, e.g. with research questions, background, methods and expected results. If in doubt how this would work, you could look at pre-registered reports for guidance:
https://www.cos.io/initiatives/prereg

The aim of the study is up to you, but it should logically follow from the scenario, and should be of personal interest to you.
It may help to consider the topics discussed in class! Or select some keyword combination: Usability, efficacy, efficiency, ethicality, safety... of pedagogy, professional support, tutoring, etc etc.

For each decision in your study design, you should not only describe it (e.g. "*I will recruit 500 secondary school students from 50 schools in a representative sample*"), but you should also justify it to the best of your ability (e.g. "*I believe N=500 will be a well-powered experiment because the effect of interest X is likely to have an effect size Y, based on its similarity to effects seen in Author et al (2019)*").

The final deliverable is a document with well-ordered sections describing the design of your study and hypothesised outcomes.

Essay.

The essay should be around 1500 words. It will be reflective and should thus be the last task of the course. For this reason, full instructions will follow after the deadline of the study design.

Requirements:

- *Select a setting:* this course, the scenario generated in your interaction with chatGPT, or both (if choosing both, the idea is to use them as contrasting reference points)
- *Write from your point of view*: From your perspective as an educational sciences expert, reflect on how AI adds or does not add value to the learning and/or teaching, aiming to be specific to your chosen setting. Avoid broad generalisations and discussions of societal impact
- *Literature Review*: incorporate existing research on the pedagogical value of AI, including but not limited to 3-5 papers or other sources taken from the course Moodle.
- *Elaborate a theme*: Incorporate ONE of the following themes in your essay, typing it in to your specific setting:

1. How could guided interactions with AI, like the one you engaged with during the course, potentially enhance learning experiences? Can you provide specific examples or scenarios where a prompting template might be useful in educational contexts?
2. Discuss the concept of "shared agency" in the context of AI in education. How can educators and AI tools work together to foster student learning? What potential problems could arise from this shared agency?
3. How might AI in education impact the role of the teacher (and/or the nature of teaching/learning)? How can teachers best adapt to this changing landscape?
4. How might the implementation of AI in education have an impact on the teacher’s task of creative guidance in the classroom? Could AI potentially limit or enhance creativity in the classroom?
5. Reflect on your own experiences in the classroom and consider how AI could have been beneficial or detrimental in that setting.

6. Epistemic confirmation (reinforcing one’s own knowledge) versus epistemic exploration (seeking out challenging new insights), might influence how one interacts with cutting-edge AI. Reflect on how this might look in a real-world classroom setting.

- *Conclude* with a concise singular argument for or against pedagogical AI, informed by your reflections above

References: list all references cited in the essay.

Topic Modeling

In addition to the standard BERTopic report, we included a count of participants mentioning each topic, along with a Shannon Entropy index to measure participant diversity. This Shannon index differs from the participants' topic diversity discussed in our article; it estimates how evenly each topic is distributed among students (i.e., topics' presence across students). In general, topics with a Shannon Index below 0.6 tend to be dominated by a single participant, while higher values indicate more balanced distribution across participants.

Supplementary Table 2. Topic Modeling of Study Designs. Identified topics in study designs and representation.

Topic	Count	Name	Representation	Participants	Shannon Index
0	277	0_ai education_ai academic_ai tutor_ai literacy	['ai education', 'ai academic', 'ai tutor', 'ai literacy', 'use ai', 'using ai', 'ai native', 'online learning', 'ai based', 'ai']	23	1.21
1	179	1_chatgpt students_chatgpt academic_experience chatgpt_uses chatgpt	['chatgpt students', 'chatgpt academic', 'experience chatgpt', 'uses chatgpt', 'using chatgpt', 'chatgpt', 'chatgpt usage', 'use chatgpt', 'role chatgpt', 'chatgpt provide']	22	1.14
2	201	2_digital learning_learners_learning environment_learning environments	['digital learning', 'learners', 'learning environment', 'learning environments', 'digital literacy', 'online learning', 'student engagement', 'analytics', 'learning platform', 'based learning']	18	0.98
3	119	3_analyses used_analysis data_analyses_participants study	['analyses used', 'analysis data', 'analyses', 'participants study', 'descriptive statistics', 'analysis descriptive', 'data analysis', 'statistics', 'analyzed using', 'data analyzed']	23	1.25
4	74	4_self assessments_students psychological_study related_intervention course	['self assessments', 'students psychological', 'study related', 'intervention course', 'course intervention', 'assessments', 'participating students', 'sample students', 'adaptive intervention', 'intervention group']	21	0.96
5	57	5_creativity tests_assessing creativity_creativity results_creativity matriculation	['creativity tests', 'assessing creativity', 'creativity results', 'creativity matriculation', 'students creative', 'creativity', 'defining creativity', 'thinking assessment', 'creativity article', 'competences pedagogical']	6	0.25
6	60	6_professional development_teacher professional_teacher pd_teachers professional	['professional development', 'teacher professional', 'teacher pd', 'teachers professional', 'teachers educational', 'teacher learning', 'professional learning', 'educational leaders', 'teachers', 'supporting teacher']	8	0.57

7	45	7_learning disabilities_disabled students_education disability_students disabilities	['learning disabilities', 'disabled students', 'education disability', 'students disabilities', 'disabilities', 'learning outcomes', 'english learning', 'disabilities disability', 'skills disabled', 'autonomy competence']	11	0.49
8	41	8_image generators_image generating_generated images_ai image	['image generators', 'image generating', 'generated images', 'ai image', 'image generation', 'ai generated', 'image critically', 'generative ai', 'images', 'media literacy']	3	0.18
9	34	9_2021_kendall 2016_2019_courses 2018	['2021', 'kendall 2016', '2019', 'courses 2018', '2018', 'education 2017', '2020', 'educ 2012', 'education 2020', 'magnus 2018']	11	0.89
10	46	10_reading skills_reading ability_reading children_children reading	['reading skills', 'reading ability', 'reading children', 'children reading', 'reading achievement', 'improve reading', 'personalized reading', 'individualized reading', 'students reading', 'reading motivation']	4	0.18
11	30	11_learning neurotechnology_use neurotechnology_neurotechnology education_neurotechnology improve	['learning neurotechnology', 'use neurotechnology', 'neurotechnology education', 'neurotechnology improve', 'neurotechnology enhanced', 'neurotechnology enhance', 'neurotechnology individual', 'neurotechnology used', 'neurotechnology', 'neurotechnology contribute']	5	0.56
12	28	12_picture books_picture book_books child_child mathematical	['picture books', 'picture book', 'books child', 'child mathematical', 'matching books', 'books based', 'child language', 'books suitable', 'filter books', 'books according']	3	0.3
13	29	13_consent study_study consent_informed consent_consent participate	['consent study', 'study consent', 'informed consent', 'consent participate', 'individuals consent', 'consent obtained', 'consent', 'participate study', 'pictorial consent', 'participation study']	13	0.99
14	31	14_eeg headsets_eeg headbands_brain activity_eye tracking	['eeg headsets', 'eeg headbands', 'brain activity', 'eye tracking', 'neural activity', 'monitors voice', 'respiration monitors', 'brainwave activity', 'eeg', 'movements physiological']	7	0.65
15	24	15_academic writing_students writing_writing skills_writing	['academic writing', 'students writing', 'writing skills', 'writing', 'academic texts', 'improvement academic', 'skills academic', 'skills linguistic', 'language proficiency', 'studies skills']	11	0.9
16	49	16_researchers_research_future research_results study	['researchers', 'research', 'future research', 'results study', 'planned research', 'results discussion', 'impacts study', 'study significant', 'phases study', 'innovat']	9	0.8

17	31	17_activity cognitive_cognitive processes_processes educational_neural activity	['activity cognitive', 'cognitive processes', 'processes educational', 'neural activity', 'educational technologies', 'cognitive', 'spent learning', 'learning process', 'motivation mechanisms', 'effective educational']	12	0.86
18	22	18_research ethics_research integrity_ethical considerations_ethical review	['research ethics', 'research integrity', 'ethical considerations', 'ethical review', 'ethical guidelines', 'ethics review', 'ethical concerns', 'integrity ethical', 'ethical', 'ethics']	10	0.95
19	41	19_exams_final exam_exam_matriculation exam	['exams', 'final exam', 'exam', 'matriculation exam', 'marks final', '20 marks', '30 marks', 'marks 10', 'tests', 'marks']	6	0.42
20	23	20_data privacy_privacy data_data anonymized_privacy research	['data privacy', 'privacy data', 'data anonymized', 'privacy research', 'sensitive data', 'privacy test', 'privacy maintained', 'privacy', 'data protection', 'anonymizing']	13	0.94
21	22	21_study groups_study group_groups use_groups	['study groups', 'study group', 'groups use', 'groups', 'participants groups', 'group level', 'groups provide', 'groups activity', 'groups control', 'group participants']	8	0.69
22	17	22_multiple intelligences_intelligences assessed_intelligences theory_intelligences assessment	['multiple intelligences', 'intelligences assessed', 'intelligences theory', 'intelligences assessment', 'intelligences widely', 'intelligences intelligence', 'intelligence assessment', 'intelligences children', 'intelligence interpersonal', 'intelligence measured']	6	0.44
23	16	23_home economics_economics education_economics teaching_economics school	['home economics', 'economics education', 'economics teaching', 'economics school', 'economics subject', 'economics', 'education', 'subject ai', 'tasks home', 'home ecological']	2	0.1
24	23	24_educational practices_issues knowledge_evidence tested_knowledge facilitate	['educational practices', 'issues knowledge', 'evidence tested', 'knowledge facilitate', 'evidence based', 'evidence available', 'implementation findings', 'justification educational', 'evidence centered', 'inform educational']	5	0.54
25	14	25_teaching equality_culturally responsive_teacher culturally_responsive teaching	['teaching equality', 'culturally responsive', 'teacher culturally', 'responsive teaching', 'equality competence', 'teaching gay', 'teaching education', 'teaching', 'teaching meaningful', 'teaching school']	6	0.48
26	16	26_rainforests brainstorm_ecosystems_ecosystem_ai generated	['rainforests brainstorm', 'ecosystems', 'ecosystem', 'ai generated', 'exploring rainforest', 'realisticness ai', 'like rainforests', 'environment critical', 'environments learning', 'insights subject']	3	0.22

27	16	27_child development_childhood education_learning kindergarten_early childhood	['child development', 'childhood education', 'learning kindergarten', 'early childhood', 'curriculum early', 'core curriculum', 'education child', 'finland child', 'education criteria', 'kindergarten']	4	0.41
28	18	28_finnish phd_helsinki research_university helsinki_survey finnish	['finnish phd', 'helsinki research', 'university helsinki', 'survey finnish', 'professionals finland', 'uni helsinki', 'helsinki hitp', 'helsinki', 'finland', 'survey data']	8	0.81
29	32	29_supervised machine_machine learning_supervised_supervised learning	['supervised machine', 'machine learning', 'supervised', 'supervised learning', 'classification', 'item training', 'random forest', 'learning model', 'model training', 'training machine']	6	0.63
30	17	30_learning curiosity_measure curiosity_curiosity measured_curiosity	['learning curiosity', 'measure curiosity', 'curiosity measured', 'curiosity', 'intervention curiosity', 'stretch curiosity', 'interests enhances', 'motivation enthusiasm', 'adolescent', 'seek information']	8	0.73
31	26	31_higher education_studies higher_increased academic_education 19	['higher education', 'studies higher', 'increased academic', 'education 19', 'female students', 'students', 'number students', 'educational psychology', 'education 2009', 'education']	10	0.93
32	19	32_research dbr_dbr methodology_study implemented_research design	['research dbr', 'dbr methodology', 'study implemented', 'research design', 'method research', 'based research', 'methodology research', 'dbr', 'methodology valuable', 'study method']	12	0.92
33	18	33_reading children_reading preschool_children reading_personalized reading	['reading children', 'reading preschool', 'children reading', 'personalized reading', 'knowledge children', 'experiences preschool', 'methods children', 'reading', 'based children', 'preschool children']	7	0.59
34	17	34_gt theories_theory gt_theory writing_theories data	['gt theories', 'theory gt', 'theory writing', 'theories data', 'constructing theory', 'theory using', 'theories', 'construct theories', 'theories constructed', 'gt researcher']	3	0.38

Supplementary Table 3. Topic Modeling of Essays. Identified topics in essays and representation.

Topic	Count	Name	Representation	Participants	Shannon Index
0	233	0_ai education_ai teaching_ai teachers_teachers ai	['ai education', 'ai teaching', 'ai teachers', 'teachers ai', 'use ai', 'using ai', 'development ai', 'intelligence teaching', 'ai', 'value ai']	20	1.23
1	194	1_learning environments_teacher learning_performance students_individual learning	['learning environments', 'teacher learning', 'performance students', 'individual learning', 'online courses', 'learning behavior', 'study', 'act based', 'students', 'online course']	21	1.19
2	69	2_creativity classroom_image generators_arts education_generated images	['creativity classroom', 'image generators', 'arts education', 'generated images', 'image generator', 'creativity', 'visual arts', 'creative technology', 'creating images', 'using image']	6	0.3
3	76	3_ai systems_artificial intelligence_intelligence ai_written ai	['ai systems', 'artificial intelligence', 'intelligence ai', 'written ai', 'human intelligence', 'ai literacy', 'machine learning', 'neural networks', 'computers human', 'imitate human']	20	1.16
4	91	4_ai tutors_ai tutor_students learning_student learning	['ai tutors', 'ai tutor', 'students learning', 'student learning', 'make learning', 'learning environments', 'learners', 'course ai', 'tutors', 'ai provide']	18	1.05
5	48	5_students chatgpt_use chatgpt_chatgpt_models chatgpt	['students chatgpt', 'use chatgpt', 'chatgpt', 'models chatgpt', 'limitation chatgpt', 'means chatgpt', 'highlight chatgpt', 'chat gpt', 'chatgpt time', 'texts']	13	0.95
6	52	6_emerging challenges_monitoring strategies_development_research	['emerging challenges', 'monitoring strategies', 'development', 'research', 'development work', 'researchers', 'strategies', 'technology', 'analysis', 'technological']	15	1.11
7	43	7_emotional ai_example ai_emotional learning_children emotional	['emotional ai', 'example ai', 'emotional learning', 'children emotional', 'behavior teacher', 'social skills', 'ai help', 'children social', 'ai simulations', 'children learn']	4	0.18
8	33	8_idea computational_computational thinking_ct skills_computational	['idea computational', 'computational thinking', 'ct skills', 'computational', 'programming', 'expert systems', 'programing', 'learning', 'problems programming', 'thinking skills']	7	0.62
9	37	9_interactions children_children social_kindergarten adapting_issues kindergarten	['interactions children', 'children social', 'kindergarten adapting', 'issues kindergarten', 'kindergarten environment', 'guiding child', 'help child', 'early childhood', 'kindergarten example', 'children']	4	0.34

10	28	10_ai educational_ai tutor_basics ai_chatbots virtual	['ai educational', 'ai tutor', 'basics ai', 'chatbots virtual', 'chatbots', 'chatbot', 'using chatgpt', 'virtual assistants', 'model chatgpt', 'educational']	10	0.9
11	23	11_systems thinking_climate change_learning discuss_thinking skills	['systems thinking', 'climate change', 'learning discuss', 'thinking skills', 'learners', 'climate', 'expertise development', 'individual learning', 'ecological systems', 'learning']	3	0.15
12	28	12_ai ethics_aware ethical_ethical considerations_intelligence useful	['ai ethics', 'aware ethical', 'ethical considerations', 'intelligence useful', 'ethical aspects', 'ethical', 'ethical practices', 'intelligence work', 'ethical issues', 'ethics']	11	0.96
13	24	13_school leaders_impacts principals_school principals_school heads	['school leaders', 'impacts principals', 'school principals', 'school heads', 'principals empathetic', 'principals difficulties', 'principals early', 'principals', 'principal wellbeing', 'heads teacher']	6	0.46
14	19	14_analytics education_learning analytics_predictive analytics_inform students	['analytics education', 'learning analytics', 'predictive analytics', 'inform students', 'analytics', 'prevent students', 'students', 'learners', 'instance students', 'predictive']	10	0.91
15	20	15_world deterministic_world probabilistic_world probabilities_probabilistic thinking	['world deterministic', 'world probabilistic', 'world probabilities', 'probabilistic thinking', 'world logical', 'predictions societal', 'deterministic', 'probabilistic reasoning', 'probabilistic', 'theories probabilistic']	3	0.23
16	17	16_storytelling diversity_diversity trainings_diversity intervention_diversity inclusion	['storytelling diversity', 'diversity trainings', 'diversity intervention', 'diversity inclusion', 'storytelling projects', 'implementing diversity', 'diversity inclu', 'diversity', 'chatgpt powerful', 'digital diversity']	2	0.1
17	18	17_essay briefly_topic interesting_ideas article_idea write	['essay briefly', 'topic interesting', 'ideas article', 'idea write', 'inspired ideas', 'sections essay', 'perspectives sections', 'subject', 'assignment wrote', 'cited assignment']	11	0.98
18	22	18_guided interacti_guided interactions_interactions provide_interactions ai	['guided interacti', 'guided interactions', 'interactions provide', 'interactions ai', 'learning guided', 'learning experiences', 'enhance learning', 'guided approach', 'interactions prompts', 'methods guided']	8	0.75
19	20	19_conversational ai_engaging conversational_promote dialogue_facilitate collaborative	['conversational ai', 'engaging conversational', 'promote dialogue', 'facilitate collaborative', 'conversational', 'fostering collaboration', 'ai potential', 'group dialogue', 'dialogue', 'discussions']	3	0.32

20	17	20_tossell 2024_2024 silvestre_costa eb_williams breazeal	['tossell 2024', '2024 silvestre', 'costa eb', 'williams breazeal', 'scherpbier ajja', 'vleuten cpm', '2023 tossell', 'fonseca santana', 'silvestre et', 'bok 2023']	8	0.87
21	23	21_writing ai_ai students_academic writing_learning ai	['writing ai', 'ai students', 'academic writing', 'learning ai', 'generative ai', 'enhancing academic', 'improve writing', 'intelligence intention', 'writing', 'essays']	8	0.77
22	14	22_students failures_predict students_fail introductory_student prediction	['students failures', 'predict students', 'fail introductory', 'student prediction', 'students risk', 'academic failures', 'student skills', 'students', 'study effectiveness', 'students academic']	5	0.49
23	11	23_tacit knowledge_knowledge skill_teaching skill_knowledge teaching	['tacit knowledge', 'knowledge skill', 'teaching skill', 'knowledge teaching', 'knowledge', 'knowledge implicit', 'teacher tacit', 'knowledge term', 'implicit knowledge', 'practical knowledge']	3	0.37

Supplementary Table 3. Topic Modeling of Keywords. Identified topics in keywords and representation.

Topic	Count	Name	Representation	Participants	Shannon Index
0	21	0_individual learning_learning individual_digital literacy_learning	['individual learning', 'learning individual', 'digital literacy', 'learning', 'literacy digital', 'learners', 'literacy', 'learning learning', 'collaborative learning', 'enhancing learning']	17	1
1	21	1_teaching purposes_purposes teaching_purposes teachers_teaching school	['teaching purposes', 'purposes teaching', 'purposes teachers', 'teaching school', 'teaching', 'purposes lecturers', 'leaders teaching', 'teachers', 'school leaders', 'teachers teaching']	12	0.96
2	15	2_powered neurotechnologies_neurotechnologies artificial_neurotechnologies machine_neurotechnologies neural	['powered neurotechnologies', 'neurotechnologies artificial', 'neurotechnologies machine', 'neurotechnologies neural', 'neurotechnologies', 'eeg brainwave', 'brainwave patterns', 'specific brainwave', 'patterns eeg', 'neural activity']	10	0.91
3	14	3_psychological flexibility_flexibility psychological_human development_flexibility	['psychological flexibility', 'flexibility psychological', 'human development', 'flexibility', 'flexibility empathy', 'development human', 'psychological', 'development empathy', 'empathy development', 'development curiosity']	9	1.02
4	14	4_online platform_experiences online_experiences inclusion_environments online	['online platform', 'experiences online', 'experiences inclusion', 'environments online', 'platform exploring', 'platform', 'online learning', 'learning platform', 'inclusion cultural', 'platforms']	11	0.82
5	10	5_virtual agents_characters ai_agents virtual_ai powered	['virtual agents', 'characters ai', 'agents virtual', 'ai powered', 'ai', 'agents', 'tools ai', 'powered characters', 'ai tutors', 'simulation peer']	7	0.68
6	10	6_data analysis_analysis data_data analyse_analyse data	['data analysis', 'analysis data', 'data analyse', 'analyse data', 'advanced data', 'analysis', 'predictive analysis', 'analysis analysis', 'analysis predictive', 'analysis sophisticated']	5	0.83
7	8	7_university_university university__	['university', 'university university', '', '', '', '', '', '', '', '']	7	0.75
8	8	8_academic writing_writing academic_academic writting_writting academic	['academic writing', 'writing academic', 'academic writting', 'writting academic', 'academic text', 'writing writing', 'text academic', 'writing', 'academic', 'writting']	6	0.6
9	8	9_generative ai_ai generative_image generators_human generative	['generative ai', 'ai generative', 'image generators', 'human generative', 'generative', 'ai image', 'ai interactions', 'generators human', 'generators', 'ai']	5	0.76

10	7	10_phd researchers_researchers phd_researchers_researchers researchers	['phd researchers', 'researchers phd', 'researchers', 'researchers researchers', 'researchers research', 'research phd', 'research', 'phd', '', '']	6	0.59
11	7	11_climate change_sustainability challenges_change climate_project climate	['climate change', 'sustainability challenges', 'change climate', 'project climate', 'climate', 'sustainability', 'eu project', 'eu projects', 'projects eu', 'schoo sustainability']	4	0.85
12	7	12_schools 12_12 schools_12 school_high school	['schools 12', '12 schools', '12 school', 'high school', 'schools high', 'secondary classroom', 'schools', 'classroom 12', 'school higher', 'school']	7	0.76
13	7	13_chatgpt_chatgpt chatgpt_headset chatgpt_headset	['chatgpt', 'chatgpt chatgpt', 'headset chatgpt', 'headset', '', '', '', '', '', '']	6	0.67
14	7	14_children_children children_adolescents children_disabilities children	['children', 'children children', 'adolescents children', 'disabilities children', 'children intellectual', 'adolescents', 'adolescents adolescents', 'disabilities', 'intellectual disabilities', 'intellectual']	5	0.68
15	6	15_students_students students_students identifying_classroom students	['students', 'students students', 'students identifying', 'classroom students', 'students university', 'risk students', 'university classroom', 'classroom', 'university', 'identifying']	5	0.7
16	5	16_early childhood_childhood education_education early_early intervention	['early childhood', 'childhood education', 'education early', 'early intervention', 'childhood', 'education', 'intervention early', 'early', 'intervention', '']	5	0.7
17	5	17_language models_language model_model language_large language	['language models', 'language model', 'model language', 'large language', 'natural language', 'language language', 'language', 'models', 'models natural', 'models large']	5	0.7
18	5	18_work_work work_work tasks_tasks	['work', 'work work', 'work tasks', 'tasks', 'workspace work', 'assistance workspace', 'assistance', 'tasks careers', 'workspace', 'careers']	5	0.58
19	5	19_higher education_education_educational_education educational	['higher education', 'education', 'educational', 'education educational', 'educational sciences', 'educational process', 'sciences education', 'sciences', 'process', 'higher']	4	0.58
20	5	20_subjects wide_range subjects_subjects_educational settings	['subjects wide', 'range subjects', 'subjects', 'educational settings', 'cognitive sciences', 'sciences educational', 'educational', 'sciences', 'wide range', 'globally wide']	4	0.58
